# Identification, Characterization and Multi-Target Mechanism of Novel ACE Inhibitory Peptides from *Idesia polycarpa* Seed Cake Protein with Ultrasound-Assisted Extraction

**DOI:** 10.3390/molecules31173050

**Published:** 2026-08-31

**Authors:** Puchao Huang, Jin He, Jiongfu Huang, Rui Liu, Jing Zhang

**Affiliations:** 1 School of Food Science and Engineering, Guiyang University, Guiyang 550005, China; huangpc2025@163.com (P.H.);; 2 Key Laboratory of *Idesia polycarpa*, National Forestry and Grassland Administration, Guiyang 550005, China

**Keywords:** *Idesia polycarpa* meal, food-derived bioactive peptides, blood pressure regulation activity, multi-target action mechanism, molecular interaction analysis

## Abstract

Angiotensin-converting enzyme (ACE) inhibitory peptides are promising bioactive components for developing blood pressure-regulating functional foods. *Idesia polycarpa* meal (IPM), an underutilized protein-rich byproduct from oil processing, is currently restricted to low-value applications such as animal feed, and systematic research on its ACE inhibitory peptides remains largely absent. This study hypothesized that controlled enzymatic hydrolysis of IPM protein could release novel ACE inhibitory peptide candidates with high activity. Fourier-transform infrared spectroscopy and differential scanning calorimetry (DSC) confirmed that ultrasonic treatment induced moderate conformational loosening of IPM protein, with thermal denaturation temperature decreasing from 162.20 °C to 158.79 °C, while the core secondary structure remained intact, thereby improving enzymatic hydrolysis efficiency. After sequential purification via ultrafiltration and gel filtration chromatography, the CP3-H3 fraction (molecular weight < 3 kDa) exhibited the highest ACE inhibitory rate of 95.39% at 1.0 mg/mL. Amino acid analysis showed that the active fraction contained 48.92% hydrophobic amino acids and 11.55% aromatic amino acids, which matched the structural requirements for potent ACE inhibition. Eight novel ACE inhibitory peptides were identified via LC-MS/MS combined with multi-round in silico screening, all of which were not recorded in the Database of Food-derived Bioactive Peptides (DFBP). Molecular docking analysis revealed that all eight peptides could stably bind to the active pocket of ACE, among which WDW showed the strongest binding affinity to PTGS2 with a binding free energy of −10.7 kcal/mol. Network pharmacology analysis demonstrated that these peptides exerted antihypertensive effects through synergistic regulation of 142 core blood pressure homeostasis-related targets and multiple key pathways. The core peptide WNWD was chemically synthesized and verified to have an ACE inhibitory IC_50_ value of 3.529 mM. This study demonstrates that IPM is a promising food-derived source of ACE inhibitory peptides, and provides a theoretical basis for the high-value utilization of *Idesia polycarpa* processing byproducts.

## 1. Introduction

*Idesia polycarpa* Maxim (IP), a woody oil crop, generates substantial quantities of fruit meal as a byproduct after oil extraction. This agro-industrial byproduct contains approximately 30% crude protein, along with diverse bioactive constituents such as polyphenols and flavonoids (Shi et al., 2024 [1]). At present, *Idesia polycarpa* meal (IPM) is predominantly utilized as a low-value animal feed ingredient or directly discarded, resulting in severe waste of biomass and considerable environmental pressure (Zhou et al., 2022 [2]). The high-value utilization of grain and oil processing byproducts has emerged as a core research direction in contemporary food science. Numerous studies have substantiated that analogous oilseed processing residues, including soybean meal, walnut meal, and rapeseed meal, can be converted via controlled enzymatic hydrolysis into highly active angiotensin-converting enzyme (ACE) inhibitory peptides, thereby establishing a crucial nexus between byproduct valorization and functional food development. Among the proteases employed, Alcalase, an alkaline protease, is recognized as the enzyme of choice for producing food-derived bioactive peptides owing to its high hydrolytic efficiency and the pronounced ACE inhibitory activity of the resultant hydrolysates; its application has been extensively validated in the enzymatic hydrolysis of soybean protein, walnut meal protein, and rapeseed protein (Sitanggang et al., 2021; Xu et al., 2021; S. Tang et al., 2025; J. Wang et al., 2021; Meng et al., 2024; Duan et al., 2023 [3,4,5,6,7,8]). Nevertheless, current investigations into the bioactivity of IPM have been confined solely to the antioxidant properties of its crude extracts. To date, studies concerning the optimization of enzymatic hydrolysis conditions for its protein fraction, the isolation and identification of ACE inhibitory peptides, bioactivity assessment, and the elucidation of underlying molecular mechanisms remain unreported, signifying a conspicuous research gap in this domain.

Disturbances in blood pressure homeostasis represent a critical trigger for cardiometabolic health risks. According to statistical estimates, by the year 2025, the global population with abnormally elevated blood pressure approached nearly 1.56 billion, a figure substantially higher than previous projections (Islam et al., 2025 [9]). Maintaining blood pressure homeostasis and mitigating associated health risks have thus emerged as pivotal research imperatives within the fields of nutrition and food science. ACE occupies a central regulatory position within both the renin–angiotensin system (RAS) and the kallikrein–kinin system (KKS), serving as a key target for the homeostatic modulation of blood pressure in vivo (Cao et al., 2024 [10]). Consequently, the inhibition of ACE activity has been established as a core and efficacious strategy for the development of blood pressure-regulating functional components.

Although synthetic ACE inhibitors currently employed in clinical practice can rapidly modulate blood pressure, their prolonged administration is frequently associated with various adverse effects, including dry cough, dizziness, and renal impairment, thereby imposing significant limitations on their application in daily dietary interventions. With advancing research into natural bioactive constituents, food-derived ACE inhibitory peptides have garnered considerable attention as prime candidate ingredients for the development of blood pressure-regulating functional foods (Xiang et al., 2023 [11]). This is attributed to their high biosafety profile, low toxicity, favorable absorption and metabolic characteristics, and the notable absence of the common adverse effects associated with synthetic inhibitors. In comparison to synthetic ACE inhibitors, ACE inhibitory peptides derived from natural food sources not only demonstrate comparable in vitro ACE inhibitory activity but also exhibit superior dietary applicability, positioning them as high-quality functional bases for routine dietary intervention (Xue et al., 2021 [12]).

Based on the foregoing, this study proposes the hypothesis that controlled enzymatic hydrolysis of IPM protein with Alcalase can yield novel peptide sequences exhibiting high ACE inhibitory activity. These peptides are hypothesized to exert their inhibitory effects through stable binding within the active pocket of ACE and to synergistically modulate biological processes pertinent to blood pressure homeostasis via multiple targets and pathways. To validate this hypothesis, total protein was extracted from IPM and subjected to Alcalase-catalyzed hydrolysis to prepare the active hydrolysate. Fractions possessing high ACE inhibitory activity were obtained through sequential purification involving ultrafiltration and gel filtration chromatography. Peptide sequences were subsequently identified utilizing ultra-performance liquid chromatography–tandem mass spectrometry (LC-MS/MS). Furthermore, network pharmacology analysis in conjunction with molecular docking was employed to systematically elucidate the core targets and molecular interaction mechanisms underlying the identified ACE inhibitory peptides. This study aims to pioneer a novel avenue for the high-value and sustainable utilization of IPM, while concurrently providing novel natural peptide candidates and theoretical substantiation for the development of blood pressure-regulating functional foods.

## 2. Materials and Methods

### 2.1. Materials and Reagents

#### 2.1.1. Materials

Defatted cold-pressed IP meal was provided by the Key Laboratory of *Idesia polycarpa* Engineering Technology, National Forestry and Grassland Administration (Guiyang, Guizhou, China).

#### 2.1.2. Main Reagents

Alcalase (enzyme activity ≥ 200,000 U/g) was purchased from Beijing Zhongshengruitai Technology Co., Ltd. (Beijing, China). Sephadex G-25 gel was purchased from Beijing Solarbio Science & Technology Co., Ltd. (Beijing, China). *N*-[3-(2-Furyl) acryloyl]-L-phenylalanyl-glycyl-glycine (FAPGG, chromatographic purity ≥ 98%) was purchased from Shanghai Macklin Biochemical Co., Ltd. (Shanghai, China). ACE (activity ≥ 2.0 U/mg) was purchased from Shandong Keyuan Biotechnology Co., Ltd. (Jinan, Shandong, China). Simulated Gastric Fluid (SGF, compliant with INFOGEST static in vitro digestion guidelines, pepsin activity ≥ 2000 U/mL) was purchased from Beijing Leagene Biotechnology Co., Ltd. (Beijing, China). Simulated Intestinal Fluid (SIF, compliant with INFOGEST static in vitro digestion guidelines, trypsin activity ≥ 1000 U/mL) was purchased from Beijing Leagene Biotechnology Co., Ltd. (Beijing, China).

### 2.2. Preparation of Idesia polycarpa Meal Protein

IPM was pulverized using a high-speed universal grinder (FW100, Tianjin Taisite Instrument Co., Ltd., Tianjin, China) and subsequently passed through a 120-mesh sieve. The resulting powder was defatted with petroleum ether (boiling range, 60–90 °C) at a solid-to-liquid ratio of 1:5 (*w*/*v*, where w represents the dry weight of the seed cake in g and v represents the solvent volume in mL; the same notation applies hereafter) for 3 h at room temperature (20 °C). This defatting procedure was repeated twice. The filter cake was air-dried to remove residual solvent, and the petroleum ether was recovered by rotary evaporation using a rotary evaporator (RE-52AA, Shanghai Yarong Biochemical Instrument Factory, Shanghai, China). The defatted meal was then mixed with 0.08 mol/L of NaOH solution at a solid-to-liquid ratio of 1:58 (*w*/*v*) and subjected to ultrasonic extraction using an ultrasonic processor (SCIENTZ-IID, Ningbo Scientz Biotechnology Co., Ltd., Ningbo, China) under parameters optimized via preliminary experiments (20 kHz, 275 W, 50% duty cycle, 60 °C) for 90 min. The resulting suspension was centrifuged at 5000× *g* for 20 min using a high-speed refrigerated centrifuge (5810R, Eppendorf AG, Hamburg, Germany), and the supernatant was collected. The pH of the supernatant was adjusted to 3.2 (the isoelectric point of *Idesia polycarpa* meal protein (IPMP)) using 1 mol/L of HCl, and the mixture was allowed to stand at 20 °C for 4 h to facilitate precipitation. Following centrifugation at 5000× *g* for 20 min, the precipitated protein was collected, washed, freeze-dried under vacuum using a vacuum freeze dryer (LGJ-10, Beijing Songyuan Huaxing Technology Development Co., Ltd., Beijing, China), and stored in a sealed container at −20 °C protected from light until further use. For comparison, conventional alkaline-extracted protein (control group) was prepared following the identical procedure described above, except that the ultrasonic treatment step was replaced with constant-temperature water bath extraction at 60 °C for 90 min with static incubation. The protein content of the obtained IPMP was determined to be (30.37 ± 1.24)% (dry basis) via the Kjeldahl method (conversion factor: 6.25), meeting the purity requirements for subsequent enzymatic hydrolysis experiments.

### 2.3. Preparation of Enzymatic Hydrolysate

IPMP isolate was dissolved in deionized water to obtain a 1% (*w*/*v*) protein solution. The pH of the solution was adjusted to 8.0, and the mixture was pre-incubated at 55 °C for 15 min. Alcalase was subsequently added at an enzyme-to-substrate ratio of 4000 U/g. These enzymatic hydrolysis conditions were optimized through single-factor experiments combined with the Box–Behnken response surface methodology, using the in vitro ACE inhibitory rate of the hydrolysate as the primary response variable and the peptide yield as the auxiliary reference index, and were confirmed to be optimal. During hydrolysis, the pH was maintained at 8.0 ± 0.05 by continuous titration of 1 mol/L of NaOH using the pH-stat method, and the peptide yield was monitored simultaneously. The reaction was allowed to proceed at a constant temperature of 55 °C for 2.9 h. The enzyme was inactivated by placing the reaction vessel in a boiling water bath for 10 min. After cooling to room temperature, the hydrolysate was centrifuged at 10,000× *g* for 20 min at 4 °C. The resulting supernatant, designated as the ACE inhibitory peptide hydrolysate (CP), was stored at 4 °C for subsequent analysis.

### 2.4. Fourier-Transform Infrared Spectroscopy 

The extracted IPM was mixed with potassium bromide (KBr) at a mass ratio of 1:100 in an agate mortar and ground thoroughly until the particle size was less than 2 μm, ensuring a homogeneous mixture. The mixture was then pressed into a uniform, transparent pellet using a hydraulic press. Fourier-transform infrared (FTIR) spectra were recorded using an FTIR spectrometer (Nicolet iS50, Thermo Fisher Scientific, Waltham, MA, USA). Dry air was employed as background reference. Spectra were acquired over the wavenumber range of 4000 to 400 cm^−1^ at a resolution of 4 cm^−1^ with 64 scans per sample. The transmittance (%T) spectra was recorded.

### 2.5. Differential Scanning Calorimetry

An accurately weighed amount (3–5 mg, precise to 0.01 mg) of IPM sample was placed in a sealed aluminum crucible. An empty aluminum crucible of identical specifications was used as the reference. The crucible was placed in the sample cell of a differential scanning calorimeter (DSC 214 Polyma, Netzsch Group, Selb, Bavaria, Germany). Under a dynamic protective atmosphere of high-purity nitrogen (flow rate: 30 mL/min), the sample was heated from 20 °C to 400 °C at a constant heating rate of 10 °C/min. The heat flow versus temperature curve was recorded in real time.

### 2.6. Isolation, Purification, and Sequence Identification of ACE Inhibitory Peptides

#### 2.6.1. Ultrafiltration Fractionation

The crude hydrolysate (CP) was initially filtered through a 0.45 μm microporous membrane (Millipore, Burlington, MA, USA) to remove insoluble impurities. The filtrate was subsequently subjected to sequential ultrafiltration using Millipore Amicon^®^ Ultra centrifugal filter units with nominal molecular weight cut-offs (MWCOs) of 10 kDa and 3 kDa. Centrifugation was performed at 4500× *g* for 20 min at 25 °C. Fractions corresponding to molecular weight ranges of Mw > 10 kDa (CP1), 3 kDa < Mw < 10 kDa (CP2), and Mw < 3 kDa (CP3) were collected separately. Each fraction was lyophilized, and their respective ACE inhibitory activities were assessed. The <3 kDa fraction (designated CP3), which exhibited the highest inhibitory activity, was selected for further purification.

#### 2.6.2. Purification by Gel Filtration Chromatography (GFC)

Purification was conducted using a chromatographic column (1.5 cm inner diameter, 50 cm length) packed with Sephadex G-25 gel (Beijing Solarbio Science & Technology Co., Ltd., Beijing, China) to a bed height of 33 cm, yielding a bed volume of approximately 58.3 mL. The column was packed via the wet method and equilibrated with 2–3 column volumes of deionized water at a flow rate of 0.8 mL/min. Column efficiency was verified using molecular weight standards. The CP3 fraction was prepared as a 20 mg/mL solution, filtered, and loaded onto the column. The sample volume was 2.5 mL, corresponding to ≤ 5% of the total bed volume. Isocratic elution was carried out with deionized water at a flow rate of 0.8 mL/min. The eluate was monitored online at 280 nm, and fractions of 3 mL per tube were collected. Fractions corresponding to the same elution peak were pooled, lyophilized, and assayed for ACE inhibitory activity. The single fraction exhibiting the highest activity (CP3-H3) was selected for subsequent sequence identification by LC-MS/MS.

#### 2.6.3. In Vitro Simulated Gastrointestinal Digestion

The in vitro simulated gastrointestinal digestion was performed using the static in vitro digestion method from Mohammadi et al. (2023) [13] with minor modifications, utilizing commercially available Simulated Gastric Fluid (SGF, containing pepsin ≥2000 U/mL) and Simulated Intestinal Fluid (SIF, containing pancreatin ≥1000 U/mL). The CP3-H3 fraction, which exhibited the highest ACE inhibitory activity, was mixed with SGF at a volume ratio of 1:1. The pH of the mixture was adjusted to 1.5, and the reaction was allowed to proceed under constant agitation at 37 °C for 2 h. Subsequently, the pH of the post-gastric digestion sample was adjusted to 6.8, after which the sample was mixed with SIF at a volume ratio of 1:1 and incubated under constant agitation at 37 °C for an additional 4 h. Upon completion of the digestion, the enzymes were inactivated by heating the sample in a boiling water bath for 10 min. The digestate was then centrifuged at 10,000× *g* for 10 min at 4 °C. The resulting supernatant was collected for the determination of ACE inhibitory activity, and the activity retention rate before and after digestion was evaluated.

#### 2.6.4. Assessment of ACE Inhibitory Peptide Activity

The ACE inhibitory activity was assessed according to the method described in Asoodeh et al., (2016) [14] with slight modifications. The assay principle is based on the ACE-catalyzed hydrolysis of the substrate *N*-[3-(2-furyl) acryloyl]-L-phenylalanyl-glycyl-glycine (FAPGG) to yield FAP and glycyl-glycine, a reaction accompanied by a decrease in absorbance at 340 nm. ACE inhibitory peptides compete with FAPGG for binding to the active site of ACE, thereby inhibiting the hydrolytic reaction. The inhibition rate is calculated based on the magnitude of the change in absorbance. All reagents were prepared using 80 mmol/L of Tris-HCl buffer (containing 0.3 mol/L of NaCl, pH 7.5). The assay was conducted in 96-well plates according to the reagent addition scheme presented in Table 1. Each sample was assayed with three biological replicates and three technical replicates. Captopril was used as a positive control. Immediately following the addition of reagents, the plate was placed in a microplate reader (Infinite M200 Pro, Tecan Group Ltd., Männedorf, Switzerland) pre-equilibrated at 37 °C, and the absorbance at 340 nm was measured at 1 min intervals over a continuous 30 min period. The ACE inhibitory activity was calculated using the following formula:

(1) $$\mathrm{ACE}\;\mathrm{inhibitory}\;\mathrm{activity}\left(\%\right)=\left(1-\frac{∆Ainhibitor}{∆Acontrol}\right)\times 100$$

where Δ*A*control represents the change in absorbance at 340 nm over 30 min in the control reaction and Δ*A*inhibitor represents the corresponding change in absorbance when the sample solution is added.

#### 2.6.5. Amino Acid Analysis

Sample pretreatment was conducted in accordance with the Chinese National Standard GB 5009.124-2016 [15], “Determination of Amino Acids in Foods.” Amino acid analysis was performed using an automatic amino acid analyzer (L-8900, Hitachi High-Technologies Corporation, Tokyo, Japan) under the following conditions: column temperature, 45 °C; reactor temperature, 115 °C; flow rate, 250 μL/min; and injection volume, 20 μL.

#### 2.6.6. Peptide Sequence Identification by LC-MS/MS

Peptide sequence identification of the fraction exhibiting the highest ACE inhibitory activity was outsourced to a professional analytical testing facility. The analytical protocol was adapted from a previously published standard method for food-derived peptide identification (Xu et al., 2021 [4]), with minor optimization of the chromatographic elution gradient and normalized collision energy to match the physicochemical properties of IPM-derived short peptides and improve identification coverage. The analysis was performed using an ultra-high-performance liquid chromatography coupled with a quadrupole electrostatic field Orbitrap mass spectrometer (Q Exactive HF-X, Thermo Fisher Scientific, Waltham, MA, USA). The chromatographic conditions were as follows: The analytical column was a NanoViper C_18_ column (75 μm × 25 cm, 2 μm particle size, 100 A pore size, Thermo Fisher Scientific, Waltham, MA, USA). Mobile phase A consisted of 0.1% formic acid in water, and mobile phase B consisted of 80% acetonitrile in water containing 0.1% formic acid. The flow rate was set to 300 nL/min, and the gradient elution program is presented in Table 2. The injection volume was 2 μL.

The mass spectrometry conditions were set as follows: The data-dependent acquisition (DDA) mode was employed. For full MS scans, the resolution was set to 60,000, the automatic gain control (AGC) target was set to 250%, and the maximum injection time was 50 ms, with a scan range of 100–1500 *m*/*z*. For MS/MS scans, the resolution was set to 15,000, the AGC target was set to 75%, the maximum injection time was 22 ms, the loop count was set to 1.0 s, and the normalized collision energy was set to 32 eV.

Database searching and peptide selection: Peptide identification was performed using MaxQuant software (version 2.4.1.0). The database employed was the IP reference protein database (taxonomy ID: 77056) downloaded from UniProt (uniprotkb-taxonomy-id-77056-2025-11-10. fasta). The search parameters were set to the “unspecific” enzyme mode. The false discovery rate (FDR) threshold was set to 1% at the peptide level, and the identification confidence level was set to ≥95%. De novo sequencing was concurrently performed to verify the accuracy of the identified peptide sequences.

### 2.7. Network Pharmacology

#### 2.7.1. Screening of Potential Bioactive Components

A total of 6083 non-redundant peptide sequences were identified from the CP3-H3 fraction via LC-MS/MS analysis. Candidate peptide screening was conducted according to the following sequential workflow: (1) The bioactivity potential of the peptides was predicted using the PeptideRanker online tool (http://bioware.ucd.ie/~compass/biowareweb/Server_pages/help/peptideranker/help.php, accessed on 3 November 2025), and sequences with predicted activity scores > 0.6 were retained. (2) Potential toxicity was assessed using ToxinPred (https://webs.iiitd.edu.in/raghava/toxinpred/index.html, accessed on 5 November 2025) based on the SVM model, and only peptides predicted to be non-toxic were retained. (3) ACE inhibitory potential was evaluated using the pLM4ACE online prediction platform (https://sqzujiduce.us-east-1.awsapprunner.com/, accessed on 8 November 2025) with the Logistic Regression model reported to have optimal performance (balanced accuracy: 0.883 ± 0.017) (Z. Du et al., 2024 [16]). The pLM4ACE platform constructs a Logistic Regression classifier based on sequence feature extraction via a protein language model, which can accurately distinguish ACE inhibitory peptides from inactive peptides by learning the sequence patterns of massive known bioactive peptides. The reported balanced accuracy of 0.883 ± 0.017 refers to the average accuracy on the independent test set after 5-fold cross-validation, which comprehensively balances the prediction performance for both positive and negative samples, indicating high reliability and generalization ability for ACE inhibitory peptide screening. (4) The water solubility and in vitro stability of the candidate peptides were predicted using the Innovagen online tool (https://www.innovagen.com/tools/, accessed on 10 November 2025) and the ExPASy ProtParam platform (https://web.expasy.org/protparam/, accessed on 10 November 2025), respectively. Peptides with favorable water solubility and a stability index <40 were retained as stable candidates. (5) Sequence alignment against the Database of Food-derived Bioactive Peptides (DFBP, http://www.cqudfbp.net/, updated December 2025, accessed on 12 November 2025) was performed to exclude previously reported ACE inhibitory peptides.

#### 2.7.2. Target Identification

The SMILES structural notations for the eight candidate peptides were generated using an online SMILES conversion tool (https://pubchem.ncbi.nlm.nih.gov/, accessed on 15 November 2025). These SMILES strings were subsequently submitted to the SwissTargetPrediction database (https://www.swisstargetprediction.ch/, accessed on 15 November 2025) with the species filter restricted to *Homo sapiens*. Putative targets with a probability score >0.5 were retained. This threshold is the official default recommended value of the SwissTargetPrediction platform, representing a 50% probability of specific binding between the ligand and target. This cutoff has been widely adopted in network pharmacology studies of food-derived bioactive peptides (Xiang et al., 2024 [17]), and can effectively balance prediction sensitivity and specificity. Following merging and deduplication, a set of peptide-specific targets was compiled.

For the acquisition of functional targets associated with blood pressure homeostasis regulation, a comprehensive search was conducted using “blood pressure homeostasis” as the core keyword across the GeneCards database (v 5.0, https://www.genecards.org/, accessed on 16 November 2025), the OMIM database (updated March 2026, https://www.omim.org/, accessed on 16 November 2025), and the Therapeutic Target Database (TTD, updated December 2025, https://db.idrblab.net/ttd/, accessed on 16 November 2025).

For the GeneCards database, targets with a relevance score ≥ 1.0 were retained; for OMIM and TTD databases, all targets matching the keyword were included. The target sets from the three databases were merged, and deduplication was performed based on official gene symbols unified by the UniProt database, finally obtaining 13,638 non-redundant blood pressure homeostasis-related targets.

#### 2.7.3. Construction and Analysis of Protein–Protein Interaction Network

The intersecting targets were imported into the STRING database (version 12.0, https://cn.string-db.org/, accessed on 17 November 2025). The species was restricted to *Homo sapiens*, and the minimum required interaction score was set to a medium confidence level of 0.4. Disconnected nodes were hidden from the network. The protein–protein interaction (PPI) data were retrieved, and the PPI network was constructed. The PPI network was constructed by importing the 142 intersection targets into the STRING 12.0 database (species: *Homo sapiens*, minimum interaction confidence: 0.4, medium confidence). The network was visualized using Cytoscape software (version 3.10.4). Core regulatory targets within the network were identified using the CytoHubba plugin based on topological parameters, including Degree, betweenness centrality, and closeness centrality.

#### 2.7.4. Construction of Bioactive Peptide–Target–Pathway Network and Identification of Key Blood Pressure-Related Targets

The eight candidate bioactive peptides, the identified core targets, and the enriched key KEGG pathways were imported into Cytoscape software (version 3.10.4) to construct a comprehensive “bioactive peptide–target–pathway” regulatory network for visualization. Through analysis of network topological parameters, the critical nodes and core pathways involved in the regulation of blood pressure homeostasis by the candidate peptides were elucidated.

#### 2.7.5. GO Functional and KEGG Pathway Enrichment

Gene Ontology (GO) functional annotation and Kyoto Encyclopedia of Genes and Genomes (KEGG) pathway enrichment analysis of the intersecting targets were performed using the DAVID 2024q4 database (https://davidbioinformatics.nih.gov/tools.jsp, accessed on 19 November 2025), with the species restricted to *Homo sapiens*. Significance thresholds were set at *p* < 0.05 and false discovery rate (FDR) < 0.05. The GO analysis encompassed three domains, biological process (BP), cellular component (CC), and molecular function (MF), thereby elucidating the potential molecular functions and regulatory pathways through which the candidate peptides exert their bioactivity.

#### 2.7.6. Molecular Docking

The molecular docking analysis was performed using the protocol of Wargasetia et al. (2021) [18] with minor modifications. The crystal structures of all eight core target proteins were retrieved from the RCSB Protein Data Bank (https://www.rcsb.org/, accessed on 20 November 2025), including *SRC* (PDB ID: 1Y57, resolution: 2.0 A), *MMP9* (PDB ID: 1GKC, resolution: 2.0 A), *AGTR1* (PDB ID: 4YAY, resolution: 2.8 A), *MMP2* (PDB ID: 7XGJ, resolution: 2.1 A), *CTSS* (PDB ID: 2FJC, resolution: 1.6 A), *ACE* (PDB ID: 1O86, resolution: 2.0 A), *CTSB* (PDB ID: 1CSB, resolution: 2.0 A), and *PTGS2* (PDB ID: 5F19, resolution: 2.8 A). Using PyMOL software (version 2.5.2), crystallographic water molecules, non-specific ligands, and redundant chains were removed. AutoDock Tools (version 1.5.7) was subsequently employed to add polar hydrogen atoms, compute Gasteiger charges, and save the protein structure in pdbqt format. The three-dimensional structures of the eight candidate peptides were constructed using ChemDraw 2024 and optimized through energy minimization with the MM2 force field. These structures were subsequently processed in the same manner to generate pdbqt-formatted ligand files. Validation of docking methodology: For the ACE receptor, its original co-crystallized ligand captopril was used as a positive control for redocking validation. The docking grid box was centered at coordinates x: 40.25, y: 35.12, z: 42.36, with dimensions of 40 A × 40 A × 40 A and a grid spacing of 0.375 A, encompassing the entire active pocket of ACE. The root-mean-square deviation (RMSD) of the redocking result was less than 2.0 A, confirming the reliability and stability of the docking protocol for ACE. For the other seven receptors (SRC, MMP9, AGTR1, MMP2, CTSS, CTSB, PTGS2), the same redocking validation was performed using their respective co-crystallized native ligands. The grid box for each receptor was set to fully cover the native ligand binding pocket, and all redocking results showed RMSD values < 2.0 A, verifying that the docking protocol adopted in this study is also applicable and reliable for other target proteins.

### 2.8. Solid-Phase Peptide Synthesis and IC_50_ Determination

The target peptide WNWD was synthesized using the standard Fmoc solid-phase peptide synthesis (SPPS) method. The crude peptide was purified by reversed-phase high-performance liquid chromatography (RP-HPLC, Agilent 1260 Infinity II, Agilent Technologies, Santa Clara, CA, USA), and its molecular weight was confirmed by electrospray ionization mass spectrometry (ESI-MS, Agilent 6460 Triple Quadrupole, Agilent Technologies, Santa Clara, CA, USA). The purity of the final synthetic peptide was determined to be >98% by analytical RP-HPLC. Deionized water and captopril were used as the negative and positive controls, respectively. The synthetic WNWD peptide was serially diluted to five concentration gradients (10.00, 3.16, 1.00, 0.32, and 0.10 mM). The ACE inhibitory rate at each concentration was measured in triplicate. The dose–response curve was plotted with peptide concentration as the *x*-axis and ACE inhibitory rate as the *y*-axis using OriginPro 2023 software. The half-maximal inhibitory concentration (IC_50_) value was calculated from the nonlinear regression fitting equation of the dose–response curve.

### 2.9. Statistical Analysis

All experiments were conducted with three independent biological replicates, each accompanied by three technical replicates. Data are presented as mean ± standard deviation (SD). Statistical analyses were performed using SPSS Statistics software (version 27.0.1). Prior to analysis, data were assessed for normality and homogeneity of variance. For data meeting the necessary assumptions, one-way analysis of variance (ANOVA) was conducted. When homogeneity of variance was confirmed, Tukey’s multiple comparison test was employed for post hoc comparisons; otherwise, Dunnett’s T3 test was applied. A *p*-value < 0.05 was considered indicative of a statistically significant difference. Graphical illustrations were generated using Origin 2024 software.

## 3. Results and Discussion

### 3.1. Fourier-Transform Infrared Spectroscopy Analysis

**Figure 1:**
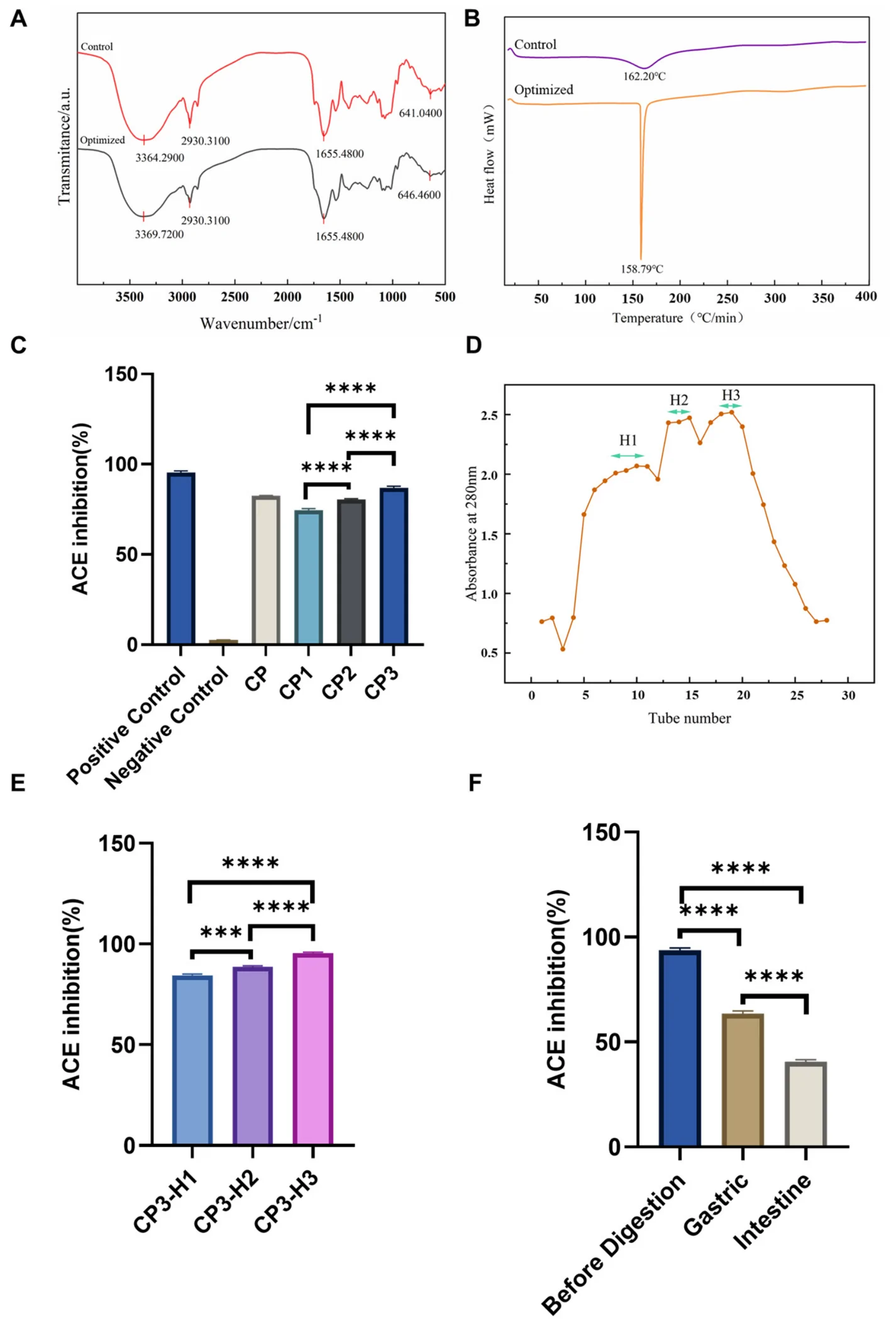
Structural characterization of ultrasound-extracted protein and isolation of gastrointestinal-stable ACE inhibitory peptide fractions from its enzymatic hydrolysate. “Control” refers to conventional alkaline-extracted protein without ultrasonic treatment, and “Optimized” refers to protein extracted by optimized ultrasound-assisted extraction. All data are expressed as mean ± standard deviation from three independent biological replicates. where *** and **** indicates an extremely significant difference between the two indicated groups (*p* < 0.001 and *p* < 0.0001). The same significance notation applies to all subsequent figures unless otherwise specified. (**A**) FTIR spectrum of the extracted protein; (**B**) DSC thermogram of the extracted protein; (**C**) ACE inhibitory rates (%) of positive control, negative control and ultrafiltration fractions; (**D**) Sephadex G-25 gel filtration elution profile of fraction CP3; (**E**) ACE inhibitory rates (%) of individual peaks from gel filtration separation; (**F**) ACE inhibitory stability of fraction CP3-H3 after in vitro simulated gastrointestinal digestion.

FTIR spectroscopy was employed to characterize the effects of ultrasound-assisted extraction (UAE) on the molecular structure of IPMP, and the results are presented in Figure 1A. Both UAE-treated and conventional alkaline-extracted protein samples exhibited characteristic infrared absorption profiles typical of plant proteins, with peak distributions highly consistent with those previously reported for rice bran and walnut meal protein isolates (H. Wang et al., 2020; L. Tang et al., 2024 [19,20]).

In the 4000–400 cm^−1^ infrared spectral range, characteristic absorption signals arise from stretching and bending vibrations of various chemical bonds in protein molecules, which effectively reflect secondary structure composition and intermolecular interactions (Lassoued et al., 2018 [21]). In the 3500–3000 cm^−1^ region, both samples displayed a broad and intense Amide A band, primarily ascribed to intramolecular and intermolecular O-H and N-H stretching vibrations. Peak position shifts and intensity changes in this region directly indicate reorganization of the hydrogen bond network. Following ultrasound treatment, the Amide A band characteristic peak blue-shifted from 3364.29 cm^−1^ to 3369.72 cm^−1^, accompanied by a significant change in peak intensity. The absorption peak at 2930.31 cm^−1^ was assigned to C-H (CH_2_, CH_3_) stretching vibrations in protein aliphatic side chains, and its intensity decreased markedly after sonication. Collectively, these observations demonstrate that ultrasonic cavitation and mechanical shear forces disrupted the native intramolecular hydrogen bond network, inducing partial protein unfolding and exposing previously buried polar moieties such as hydroxyl (-OH) and amino (-NH_2_) groups. The exposed polar groups subsequently formed a new intermolecular hydrogen bond network (Tong et al., 2022 [22]).

The Amide I band (1700–1600 cm^−1^) represents the most informative region for protein secondary structure analysis, arising predominantly from peptide bond C=O stretching vibrations with minor contributions from N-H bending vibrations (Vatansever et al., 2022; Carbonaro et al., 2012 [23,24]). Both samples exhibited Amide I band characteristic peaks at 1655.48 cm^−1^, a signature absorption of α-helical structure, with no statistically significant peak displacement detected. This finding confirms that ultrasound treatment induced only local conformational loosening and increased protein flexibility, without compromising the core secondary structure framework or causing irreversible excessive denaturation and aggregation (M. Du et al., 2018 [25]).

### 3.2. Differential Scanning Calorimetry Analysis

The DSC analysis results are presented in Figure 1B. UAE altered the thermal stability of IPMP. The thermal denaturation temperature (*T*_m_) of the untreated control protein was 162.20 °C, with a denaturation enthalpy (Δ*H*) of 18.72 J/g, whereas the *T*_m_ value of the sonicated protein decreased to 158.79 °C, with a Δ*H* of 15.36 J/g, representing a reduction of 3.41 °C in *T*_m_ and 17.9% in Δ*H*. Moreover, the endothermic denaturation peak became sharper and more symmetrical following ultrasonic treatment.

This peak shape change indicates higher structural homogeneity of protein molecules after ultrasonic treatment: ultrasound disrupted partial protein aggregates in the raw material, loosened the compact conformation, and made the thermal denaturation process more synchronous, thus presenting a sharper endothermic peak. Severe protein unfolding and random aggregation usually lead to peak broadening due to uneven denaturation temperatures of different conformations. The sharpened but not broadened peak in this study indicates that ultrasonic treatment only induced moderate and uniform conformational loosening without causing severe denaturation and irregular aggregation, which is consistent with the FTIR conclusion of the preserved core secondary structure. The decrease in Δ*H* further confirms that ultrasonic treatment weakened intramolecular non-covalent interactions and reduced protein structural stability, meaning less energy is required to complete thermal denaturation (Sun et al., 2025 [26]).

Combined with the FTIR spectral analysis results, this DSC finding supports that the cavitation effects and mechanical shearing action of ultrasound disrupt partial intramolecular non-covalent bonds, including hydrogen bonds and hydrophobic interactions, thereby inducing partial unfolding of the protein molecule and promoting a more open and relaxed spatial conformation.

### 3.3. Isolation and Purification of ACE Inhibitory Fractions

#### 3.3.1. Identification of ACE Inhibitory Activity by Ultrafiltration

Ultrafiltration is a physical sieving technique based on molecular size differences. It enables the fractionation and enrichment of enzymatic hydrolysates according to molecular weight ranges without introducing chemical reactions or compromising the structural integrity of the peptide fragments, and is therefore a commonly employed approach for the isolation and purification of food-derived bioactive peptides (Feng et al., 2019 [27]).

In the present study, the in vitro ACE inhibitory activities of the CP and its three ultrafiltration fractions—CP1, CP2, and CP3—were evaluated. Captopril served as the positive control, and ACE reaction buffer was used as the negative control. As illustrated in Figure 1C, the positive control captopril exhibited an ACE inhibitory rate of 95.82% ± 1.13% at the designated concentration, whereas the negative control displayed no significant ACE inhibitory activity, thereby validating the stability and reliability of the experimental system. The ACE inhibitory activities of the three fractionated components exhibited a pronounced molecular weight-dependent gradient: the inhibitory rates were 74.57% ± 0.49% for fraction CP1, 80.41% ± 0.32% for fraction CP2, and 86.86% ± 0.57% for fraction CP3. Notably, fraction CP3 demonstrated the highest ACE inhibitory activity, which was significantly greater than those of fractions CP1 and CP2 as well as the unfractionated CP hydrolysate (*p* < 0.05). These results indicate that peptide fragments possessing high ACE inhibitory activity are predominantly enriched in the fraction with a molecular weight below CP3. This finding is highly consistent with the results of Chen et al. (2023) [28] regarding black bean glutelin hydrolysates, which suggested that low-molecular-weight peptides generally exhibit superior ACE inhibitory activity. The underlying mechanism can be rationalized based on the structural characteristics of ACE: the active site of ACE comprises a narrow, groove-shaped hydrophobic pocket. High-molecular-weight peptides encounter substantial steric hindrance, which impedes their entry into the active pocket and subsequent binding to critical residues. In contrast, the CP3 fraction possesses greater conformational flexibility, enabling efficient access to the ACE active pocket and blockade of enzyme–substrate interactions through hydrogen bonding and hydrophobic interactions, thereby eliciting more potent inhibitory effects (Zhu et al., 2021) [29]. Furthermore, the significantly enhanced activity of fraction CP3 relative to the unfractionated crude hydrolysate confirms that ultrafiltration effectively removes high-molecular-weight unhydrolyzed proteins and incompletely hydrolyzed large peptide fragments, and achieves efficient enrichment of low-molecular-weight IPM-derived ACE inhibitory peptides, thereby laying a solid foundation for subsequent purification steps.

#### 3.3.2. Amino Acid Composition Analysis

**Figure 2:**
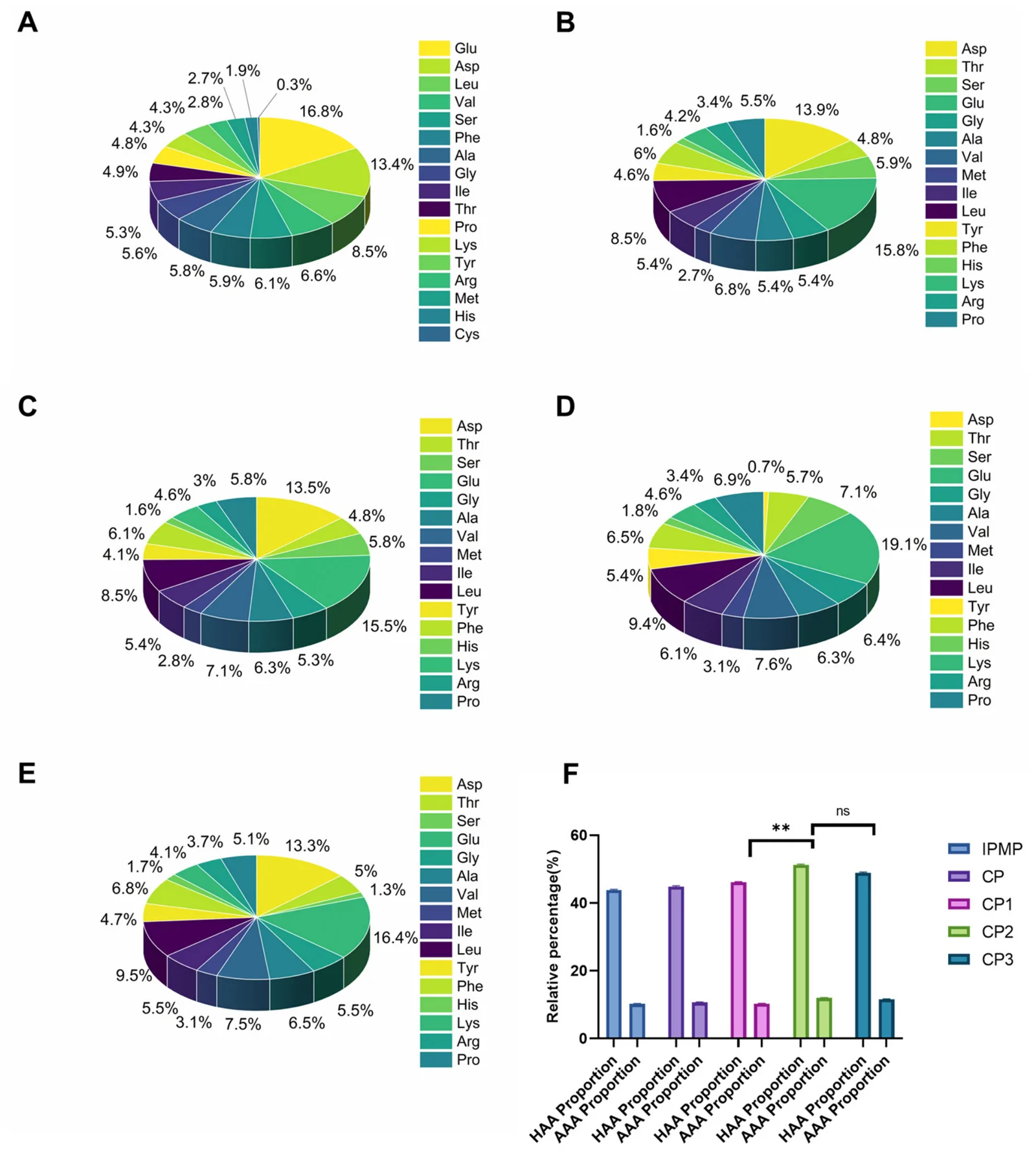
Amino acid composition of IPM raw material and its enzymatic hydrolysate fractions. (**A**) *Idesia polycarpa* meal raw material; (**B**) crude enzymatic hydrolysate (CP); (**C**) CP1 fraction (Mw > 10 kDa); (**D**) CP2 fraction (3 kDa < Mw < 10 kDa); (**E**) CP3 fraction (Mw < 3 kDa); (**F**) proportions of hydrophobic amino acids (HAAs) and aromatic amino acids (AAAs) in each ultrafiltration fraction. The symbol “**” indicates a significant difference between groups (*p* < 0.05), while “ns” indicates no significant difference between groups (*p* > 0.05).

The amino acid compositions of IPM (Figure 2A), the CP (Figure 2B), and the ultrafiltration fractions are presented in (Figure 2C–E). All fractions exhibited a comprehensive array of amino acids, with a total of 17 amino acids detected. Notably, cysteine (Cys) was undetectable in the enzymatic hydrolysate and the three ultrafiltration fractions. Previous studies have established that peptides enriched in hydrophobic and aromatic amino acids exhibit a greater propensity to bind to the active site of ACE (S. Tang et al., 2025 [5]), thereby exerting potent inhibitory effects. In the present study, the CP3 fraction (Figure 2F) contained 48.92% hydrophobic amino acids and 11.55% aromatic amino acids. Statistical analysis showed that there was no significant difference in the proportion of hydrophobic amino acids between CP2 (51.27%) and CP3 (48.92%) (*p* > 0.05). The higher ACE inhibitory activity of CP3 may be mainly attributed to its smaller molecular weight, which is more conducive to entering the ACE active pocket to exert an inhibitory effect, rather than solely depending on the proportion of hydrophobic amino acids. Among these, the contents of leucine (Leu, 20.81 mg/g), valine (Val, 16.52 mg/g), phenylalanine (Phe, 14.97 mg/g), tyrosine (Tyr, 10.38 mg/g), and proline (Pro, 11.27 mg/g) were particularly prominent.

From the perspective of structure–activity relationships, the active site of ACE comprises dual hydrophobic pockets, designated S1 and S2, situated around the Zn^2+^ center. The interior walls of these pockets are rich in hydrophobic residues, including Leu358 and Trp274 (Xiang et al., 2023 [11]). The hydrophobic side chains of Leu and Val can be accommodated within these pockets, thereby reducing the free energy of peptide–enzyme binding. The aromatic rings of Phe and Tyr can engage in π-π stacking interactions with residues in the ACE active site, and the phenolic hydroxyl group of Tyr can additionally form hydrogen bonds with Asn273 (Zhao et al., 2019 [30]). Furthermore, the pyrrolidine ring of Pro can be inserted into the S2 pocket to stabilize the peptide conformation. The high abundance of these specific amino acids constitutes the core structural foundation underlying the superior ACE inhibitory activity observed in the CP3 fraction.

#### 3.3.3. Identification of ACE Inhibitory Activity by Sephadex Gel Filtration Chromatography

Gel filtration chromatography (GFC) is a purification technique based on the molecular sieving effect. Sephadex G-25 gel is suitable for the separation and purification of small-molecule peptides. Components with larger molecular weights are unable to penetrate the gel pores and are therefore eluted first, whereas components with smaller molecular weights can enter the gel pores, resulting in a longer elution path and a later elution time (S. Tang et al., 2025 [5]).

In the present study, the CP3 fraction, which exhibited the highest ACE inhibitory activity, was further purified using a Sephadex G-25 gel filtration column. The elution profile is presented in Figure 1D. Following separation, three consecutive characteristic elution peaks were obtained from the CP3 fraction and were designated as subfractions CP3-H1, CP3-H2, and CP3-H3 according to their elution order. The ACE inhibitory activities of each subfraction were assessed at a uniform final concentration of 1.0 mg/mL. As illustrated in Figure 1E, the ACE inhibitory rates of fractions CP3-H1, CP3-H2, and CP3-H3 were 84.47% ± 0.33%, 88.65% ± 0.32%, and 95.39% ± 0.30%, respectively. Notably, the CP3-H3 subfraction, which exhibited the latest elution time and the smallest molecular weight, demonstrated significantly higher ACE inhibitory activity compared with the CP3-H1 and CP3-H2 subfractions (*p* < 0.05). This observation further corroborates that low-molecular-weight peptides constitute the principal material basis for ACE inhibitory activity. These results are consistent with the findings of Li et al. (2016) [31] regarding the isolation and purification of ACE inhibitory peptides derived from walnut meal, thereby confirming that Sephadex G-25 gel filtration chromatography enables further purification and enrichment of ACE inhibitory peptides derived from IPM. Accordingly, the CP3-H3 subfraction, which displayed the highest ACE inhibitory activity, was selected for subsequent peptide sequence identification by LC-MS/MS.

#### 3.3.4. In Vitro Simulated Gastrointestinal Digestion Stability Analysis

Oral administration represents the primary route of ingestion for food-derived bioactive peptides. The digestive stability of peptides within the gastrointestinal tract constitutes a critical prerequisite for their capacity to exert biological activity in vivo (Dang et al., 2019 [32]). In the present study, the sequential in vitro gastrointestinal digestion of the CP3-H3 fraction was conducted with reference to the INFOGEST static in vitro digestion model, utilizing commercially available SGF and SIF. The ACE inhibitory activity was measured at various stages of digestion at a uniform final concentration of 1.0 mg/mL to evaluate the digestive stability of the peptides. As illustrated in Figure 1F, the undigested CP3-H3 fraction exhibited an initial ACE inhibitory rate of 93.71% ± 0.62%. Following incubation with SGF at 37 °C for 2 h, the ACE inhibitory activity decreased significantly to 63.49% ± 0.76% (*p* < 0.05). After subsequent digestion with SIF for an additional 4 h, the activity further declined to 40.58% ± 0.59%, representing a highly significant difference compared with the undigested group (*p* < 0.01) (Meng et al., 2024 [7]). The pronounced loss of ACE inhibitory activity after gastrointestinal digestion is a common challenge faced by most food-derived bioactive peptides, which also means that the direct oral bioavailability of the peptides is limited. From the perspective of practical application, on the one hand, the peptides can still exert partial activity after digestion, and can play a sustained blood pressure regulation role through long-term dietary intake; on the other hand, targeted delivery systems such as liposome encapsulation, microemulsion embedding or enteric coating can be used to protect peptides from gastrointestinal degradation, so as to improve their bioavailability and in vivo efficacy. In addition, the degradation fragments of peptides after digestion may also have other potential biological activities, which is also a direction worthy of further exploration (Escudero et al., 2014 [33]).

### 3.4. Peptide Sequence Identification Results by LC-MS/MS

**Figure 3:**
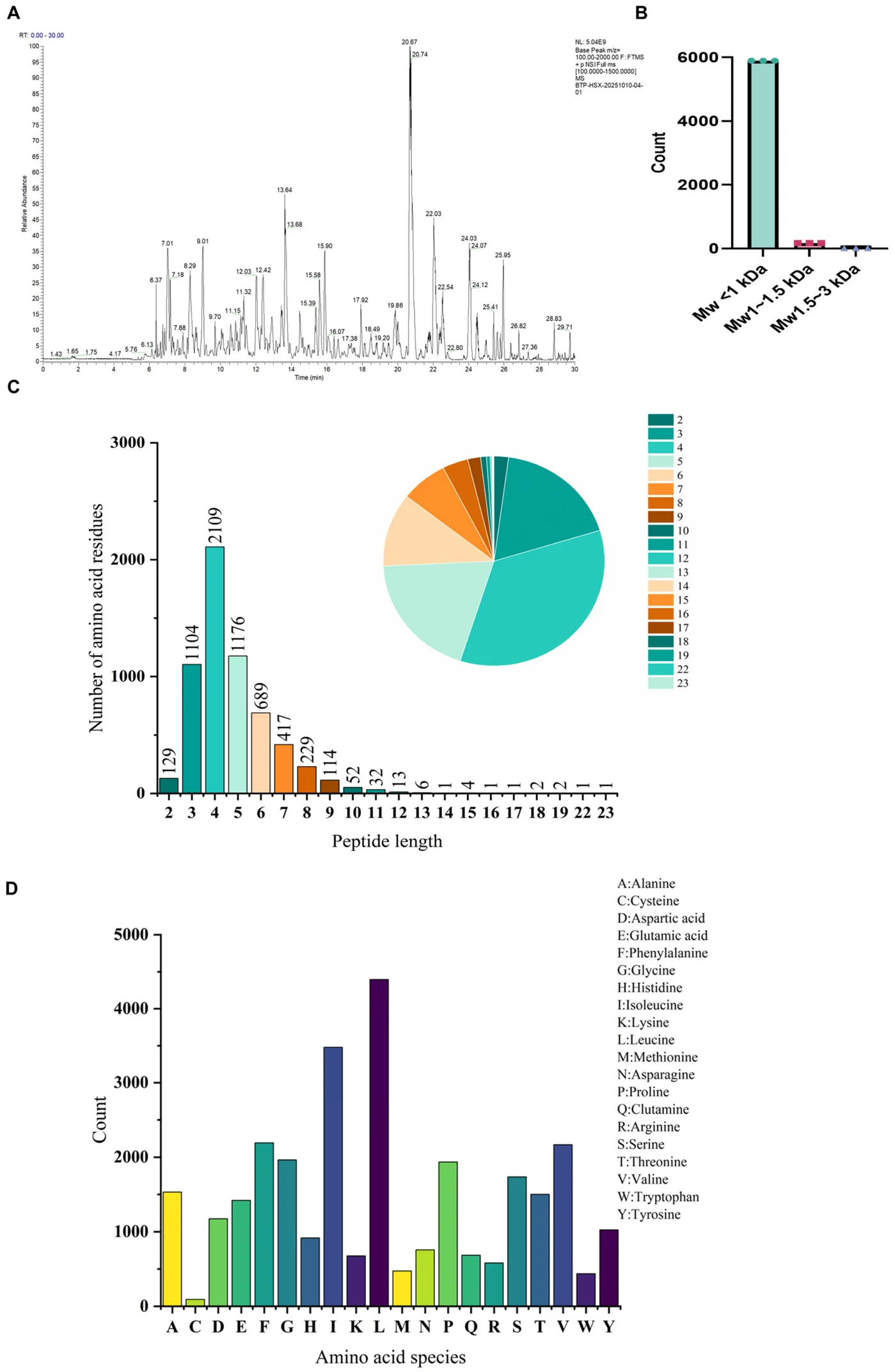
Sequence identification of ACE inhibitory peptides derived from IPMP. (**A**) MS base peak chromatogram of the CP3-H3 fraction obtained by LC-MS/MS; (**B**) peptide length distribution based on amino acid residue number of identified sequences; (**C**) molecular weight distribution of identified peptides; (**D**) amino acid composition profile of identified peptides based on residue counts.

The CP3-H3 fraction was subjected to LC-MS/MS analysis, yielding a total of 6083 non-redundant peptide sequences, as shown in Figure 3A. Molecular weight distribution analysis revealed that over 95% of the identified peptides possessed molecular weights below 1 kDa (Figure 3B). The peptide lengths ranged from 2 to 23 amino acid residues, with short peptides consisting of 2–8 residues predominating (Figure 3C), a characteristic that is highly consistent with the structural features of previously reported ACE inhibitory peptides, which are predominantly short-chain peptides comprising 2–12 amino acid residues (Xue et al., 2021 [12]).

Amino acid composition analysis of the identified peptides (Figure 3D) suggested that hydrophobic amino acids accounted for approximately 65% of the total residues. Among these, isoleucine (Ile, 14.5%), leucine (Leu, 18.3%), phenylalanine (Phe, 9.1%), and valine (Val, 9.0%) were particularly abundant, exhibiting a high degree of complementarity with the hydrophobic characteristics of the ACE active pocket. Previous studies have established that peptide chains enriched in hydrophobic residues can efficiently bind to the critical S1 and S2′ hydrophobic subsites of ACE via hydrophobic interactions, thereby competitively blocking substrate hydrolysis and exerting ACE inhibitory activity (Martin & Deussen, 2019 [34]).

Further analysis of key residues associated with ACE inhibitory activity showed that the combined proportion of aromatic amino acids—phenylalanine (Phe, 9.1%) and tyrosine (Tyr, 4.3%)—and the positively charged lysine (Lys, 2.8%) reached 16.2%. These residues constitute the core C-terminal characteristic residues that govern peptide–ACE binding affinity. Notably, Phe, recognized as a predominant C-terminal residue in ACE inhibitory peptides, can penetrate deeply into the S1′ hydrophobic pocket of ACE to form stable interactions, thereby providing a crucial structural foundation for high inhibitory potency (Lunow et al., 2015 [35]). Additionally, proline (Pro) residues accounted for 8.0% of the total amino acid composition. The distinctive cyclic structure of Pro confers a rigid turn conformation to the peptide backbone, reducing entropic penalties during binding to ACE and consequently enhancing both the binding stability and the inhibitory efficacy of the peptides.

Compared with previously reported plant-derived ACE inhibitory peptides from soybean, walnut and rapeseed, the peptides identified in this study are also dominated by short peptides with 2–8 amino acid residues and are rich in hydrophobic and aromatic amino acids, which conforms to the general structure–activity rule of food-derived ACE inhibitory peptides. However, the specific sequences of the eight peptides have not been reported in previous plant source studies, which enriches the sequence library of plant-derived ACE inhibitory peptides.

### 3.5. Screening of Candidate ACE Inhibitory Peptides from the CP3-H3 Fraction

**Figure 4:**
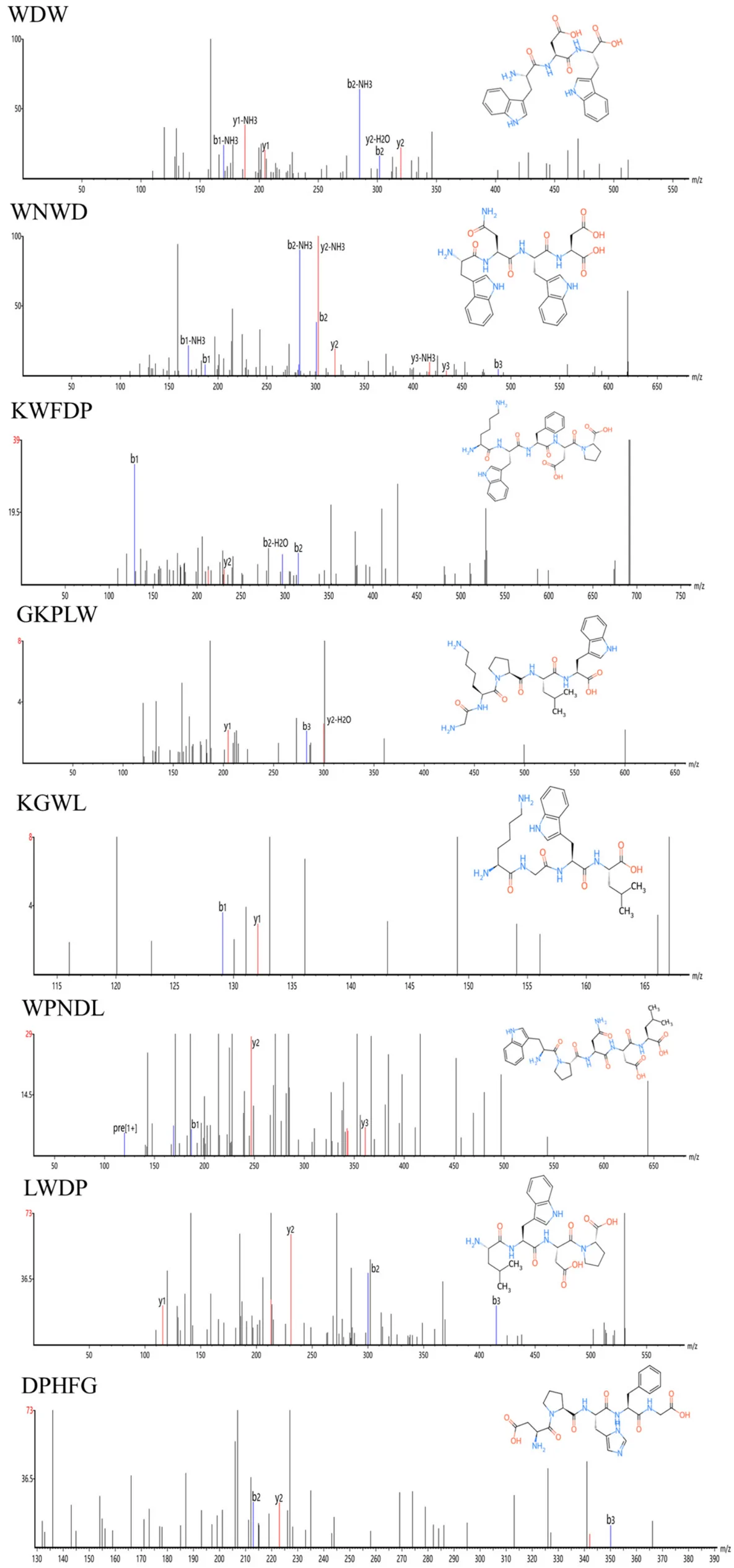
2D structures and MS/MS spectra of the eight potential antihypertensive peptides.

Based on the 6083 non-redundant peptide sequences identified in the CP3-H3 fraction via LC-MS/MS analysis, sequential virtual screening was conducted through multiple rounds of bioinformatics analysis. The workflow and corresponding results are described as follows: The bioactivity potential of the peptides was predicted using the PeptideRanker online server (with scores approaching 1 indicating higher bioactivity potential). Applying a threshold score >0.6, a total of 1523 peptide sequences with high bioactivity potential were retained. Subsequent toxicity prediction was performed using the ToxinPred online server, and only peptides predicted to be non-toxic were retained. Peptides with high ACE inhibitory activity were further screened using the pLM4ACE online prediction platform (Z. Du et al., 2024 [16]), yielding 630 candidate peptides. Water solubility and stability index predictions were then conducted using the Innovagen online tool (https://www.innovagen.com/, accessed on 25 November 2025) and the ExPASy ProtParam platform (https://web.expasy.org/protparam/, accessed on 25 November 2025), respectively; peptides exhibiting favorable water solubility and a stability index < 40 were selected as stable candidates. Finally, sequence alignment against the Database of Food-derived Bioactive Peptides (DFBP) was performed to exclude previously reported sequences. After further screening based on water solubility and in vitro stability, eight novel, previously unreported ACE inhibitory peptides were finally identified (Table 3). The corresponding MS/MS spectra and two-dimensional structural visualizations of these peptides are presented in Figure 4.

### 3.6. Network Pharmacology Analysis

#### 3.6.1. Identification of Blood Pressure-Related Potential Targets

**Figure 5:**
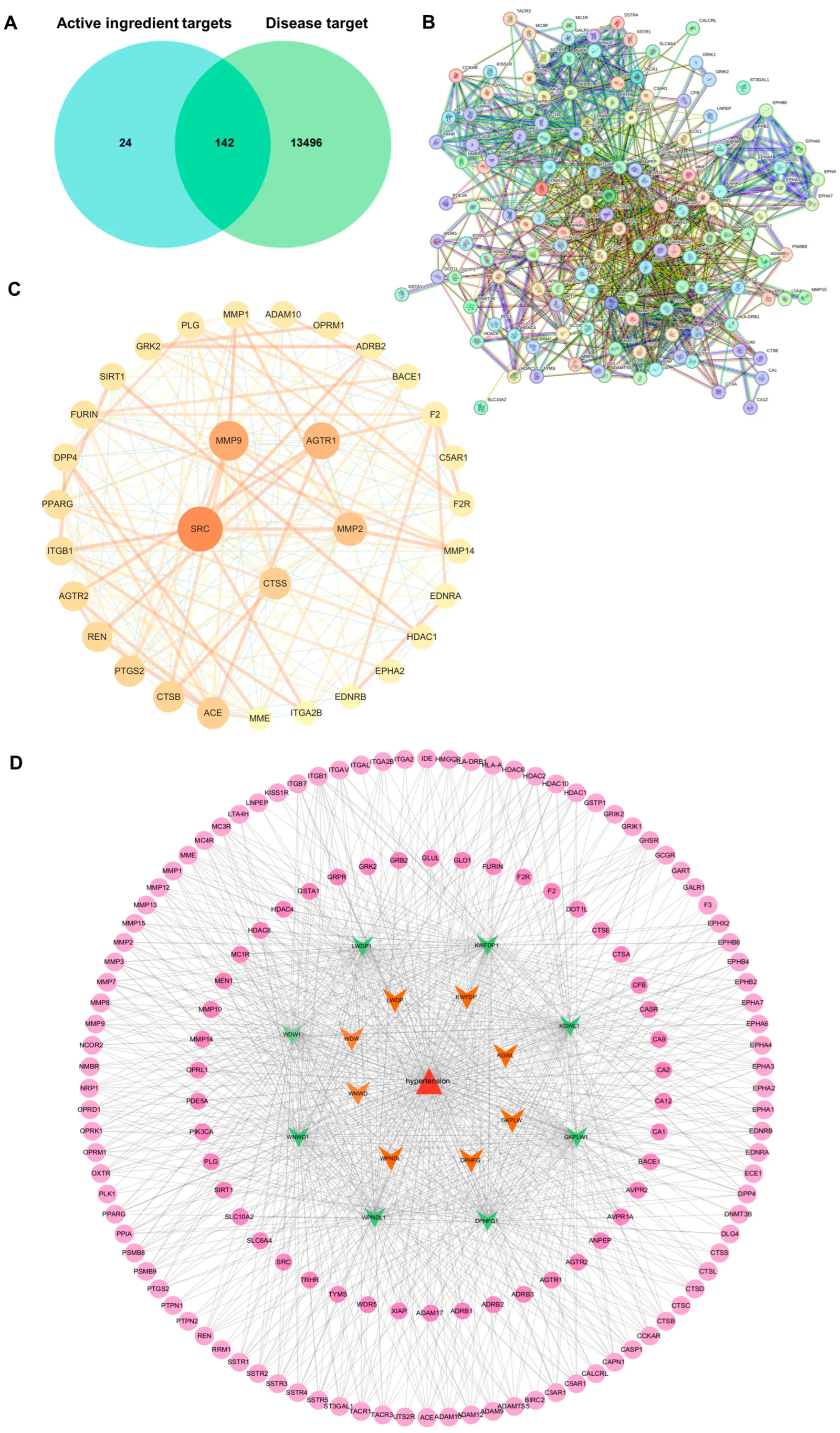
Network pharmacology analysis. (**A**) Venn diagram showing the intersection between potential targets of CP3-H3 peptides and antihypertensive targets; (**B**) PPI network of intersecting antihypertensive targets for CP3-H3 peptides; (**C**) interaction network of core targets; (**D**) integrated peptide–target–disease–pathway network for CP3-H3 peptides.

Potential targets of the eight identified peptides were predicted using the SwissTargetPrediction database (species limited to *Homo sapiens*, probability score > 0.5), yielding 166 non-redundant targets. Concurrently, 13,638 targets associated with blood pressure homeostasis were retrieved from the GeneCards, OMIM, and TTD databases using the keyword “blood pressure homeostasis”. The intersection of these two datasets identified 142 common targets (Figure 5A), accounting for 85.5% of the total predicted peptide targets. It should be noted that the blood pressure homeostasis target set covers a wide range of genes involved in vascular function, neuroregulation, endocrine metabolism and other processes, which may contribute to the high overlap ratio to a certain extent. However, the core targets screened by topological analysis are all key nodes directly related to blood pressure regulation such as RAAS, vascular remodeling and inflammatory response, rather than non-specific broad-spectrum genes, indicating that the antihypertensive effect of IPM-derived peptides has definite biological specificity rather than random coincidence. After removing two isolated nodes, the final network contained 140 nodes and 1006 edges (Figure 5B). The network exhibited dense node connections and rich interactions, with an average node degree of 14.37, which was significantly higher than that of random networks. This indicated that the antihypertensive effect of the ACE inhibitory peptides was not dependent on a single target but was achieved through synergistic interactions among multiple proteins. This network topology was consistent with previous reports on garlic-derived (Xiang et al., 2026 [36]) and grass carp swim bladder-derived (Dong et al., 2025 [37]) ACE inhibitory peptides, further confirming that food-derived antihypertensive peptides generally act via a multi-target synergistic regulatory mode, in contrast to the single-target mechanism of synthetic antihypertensive drugs (Park et al., 2023 [38]).

The top eight core regulatory targets were screened from the PPI network using the CytoHubba plugin in Cytoscape 3.10.4 with Degree value as the core topological parameter. Their biological significance in blood pressure regulation is elaborated in order of importance as follows: SRC (65), MMP9 (53), AGTR1 (47), MMP2 (40), CTSS (36), ACE (36), PTGS2 (34), and CTSB (34) (Table 4, Figure 5C). (1) SRC (Degree = 65, ranked 1st). As a core member of the non-receptor tyrosine kinase family, SRC serves as the absolute regulatory hub of the entire PPI network. It participates in the transduction of multiple blood pressure-regulation-related signaling pathways, including the PI3K/Akt/eNOS pathway, MAPK pathway, and calcium signaling pathway (Heron-Milhavet et al., 2011 [39]). Xiang et al. (2026) [36] demonstrated that ACE inhibitory peptides could promote NO production and improve endothelial function by activating the PI3K/Akt/eNOS pathway, and SRC is the key upstream regulatory molecule of this pathway. (2) MMP9 (Degree = 53, ranked 2nd) and MMP2 (Degree = 40, ranked 4th). Both are core members of the matrix metalloproteinase family, primarily responsible for degrading extracellular matrix components. Under hypertensive conditions, overexpression of MMP2/MMP9 leads to imbalance in vascular wall ECM metabolism, causing pathological changes such as vascular wall thickening and lumen stenosis (Fernandes et al., 2009; Lindsey et al., 2006 [40,41]). Dong et al. (2025) [37] confirmed that MMP9 is a core antihypertensive target of grass carp swim-bladder-derived ACE inhibitory peptides, which is highly consistent with the results of this study. (3) AGTR1 (Degree = 47, ranked 3rd) and ACE (Degree = 36, ranked 6th). These two targets are core components of the classic renin–angiotensin–aldosterone system (RAAS). ACE, as the key rate-limiting enzyme of RAAS, is the direct target of this study, providing direct target evidence for the in vitro inhibitory activity. AGTR1 is the main functional receptor of angiotensin II, and its Degree value in this study is significantly higher than that in similar studies. This suggests that IPM-derived peptides could simultaneously act on ACE and AGTR1 to achieve dual blockade of the RAAS pathway, which can more comprehensively inhibit vasoconstriction and aldosterone secretion compared with single ACE inhibition (Sowers, 2004 [42]). (4) CTSS (Degree = 36, ranked 5th) and CTSB (Degree = 34, ranked 8th). Both belong to the cathepsin family and are involved in proteolysis, directly corresponding to the “endopeptidase activity” and “peptidase activity” in the GO enrichment results (Liu et al., 2021 [43]). They participate in vascular wall remodeling and endothelial function regulation by mediating the degradation and activation of various bioactive substrates. (5) PTGS2 (Degree = 34, ranked 7th). PTGS2 (COX-2) is an inducible cyclooxygenase that mediates chronic low-grade inflammation, and hypertension has been confirmed to be a chronic low-grade inflammatory disease (Pei et al., 2022 [44]). The regulation of PTGS2 indicates that IPM-derived peptides may exert antihypertensive effects through anti-inflammatory pathways.

An integrated “active peptide–target–pathway” regulatory network was constructed by importing the eight active peptides, core targets, and significantly enriched KEGG pathways into Cytoscape, containing 159 nodes and 803 edges (Figure 5D). The results showed that all eight active peptides could simultaneously bind to multiple core targets, exhibiting multi-component, multi-target, and multi-pathway synergistic antihypertensive characteristics. Among them, ACE, as the core target of the RAAS, was the direct action site of the peptides; MMP2/MMP9 regulated extracellular matrix metabolism and participated in vascular remodeling; and PTGS2 mediated inflammatory responses and maintained vascular homeostasis (Xiang et al., 2026; Dong et al., 2025 [36,37]). This network pattern matched the “multi-target synergistic action model” of food-derived ACE inhibitory peptides from Dong et al., (2025) [37], and aligned with the “multi-pathway cascade regulation” mechanism identified in Wu et al., (2024) [45] for bovine colostrum-derived ACE inhibitory peptides, fully indicating that the antihypertensive effect of IPM peptides was a multi-level and networked regulatory result rather than simple inhibition of a single target (Rivero-Pino, 2023 [46]).

#### 3.6.2. GO Functional and KEGG Pathway Enrichment Analysis

**Figure 6:**
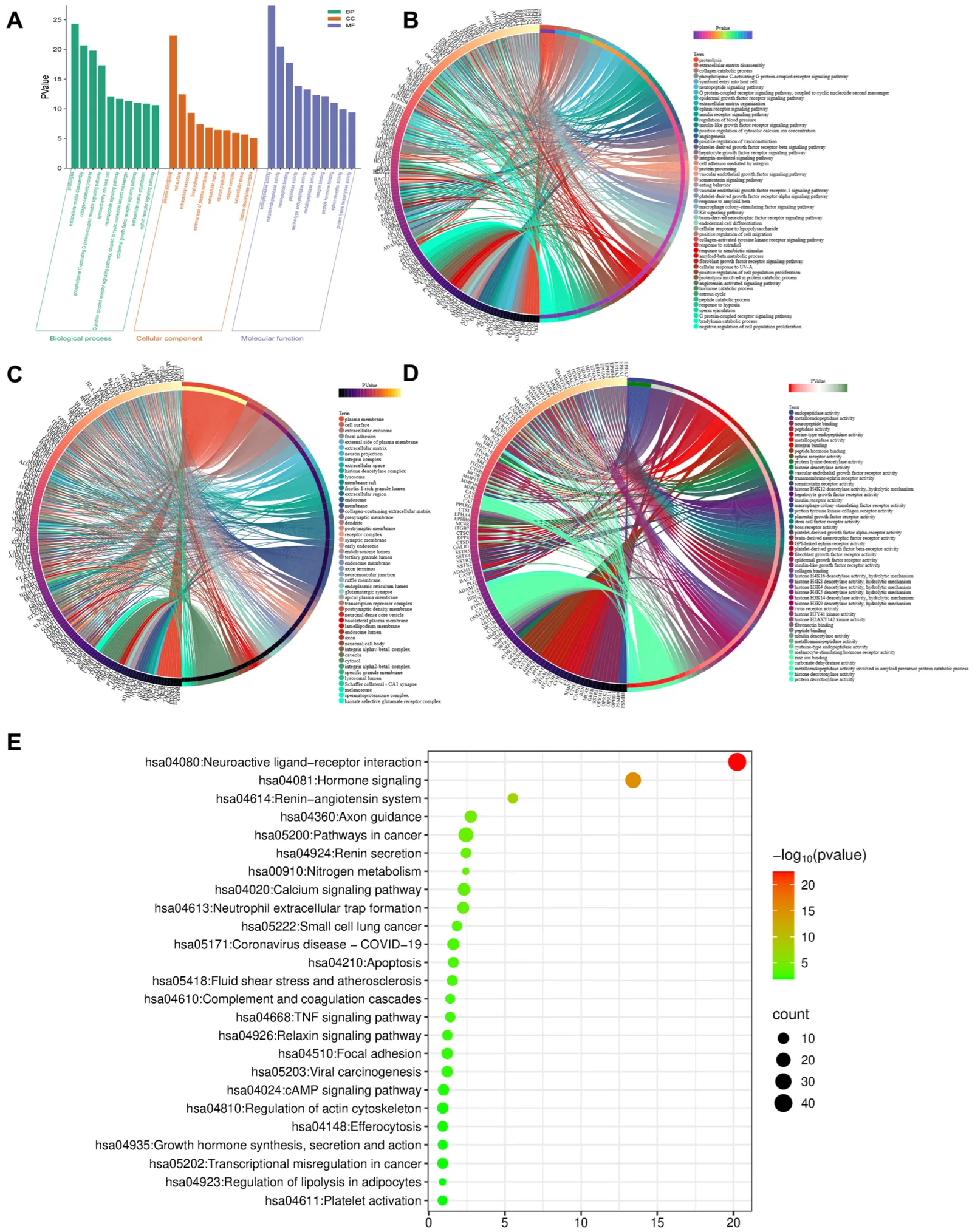
GO functional and KEGG pathway enrichment analysis. (**A**) Bar plot of GO functional enrichment for key antihypertensive targets of CP3-H3 (top 10 terms per category); (**B**) chord plot of enriched GO biological process BP terms (top 50); (**C**) chord plot of enriched GO cellular component CC terms (top 50); (**D**) chord plot of enriched GO molecular function MF terms (top 50). (**E**) Bubble plot of KEGG pathway enrichment for core antihypertensive targets of CP3-H3 (top 20 pathways).

Using *p* < 0.05 and FDR < 0.05 as screening thresholds, GO enrichment analysis of the 142 targets showed that (Figure 6A) BPs were significantly enriched in proteolysis, extracellular matrix disassembly, the collagen catabolic process, and the G protein-coupled receptor signaling pathway, which are directly related to blood pressure regulation processes such as vasoconstriction and vascular remodeling (Figure 6B); CCs were mainly localized in the plasma membrane, cell surface, and exosomes, consistent with the typical characteristic of peptides acting through membrane receptors (Figure 6C); and MFs were significantly enriched in endopeptidase activity, metalloendopeptidase activity, and peptidase activity, matching the enzymatic properties of core targets such as ACE, MMPs, and cathepsins (Figure 6D). These results aligned well with the GO enrichment profile of grass carp swim bladder peptides from Dong et al. (2025) [37] and the functional annotation of garlic antihypertensive peptides from Xiang et al. (2026) [36], further verifying the accuracy of the target functions identified in this study.

KEGG enrichment analysis revealed that the core targets were significantly enriched in pathways including neuroactive ligand–receptor interaction, the renin–angiotensin system, the calcium signaling pathway, and focal adhesion (Figure 6E). Notably, the neuroactive ligand–receptor interaction pathway was the most highly enriched (*p* < 0.001, FDR < 0.001), representing the most innovative finding of this study. Most current studies on food-derived ACE inhibitory peptides mainly focus on the classic RAAS pathway, while the involvement of neuroactive regulation pathways is rarely reported. This pathway contains various vasoactive substance receptors such as adrenergic receptors, dopamine receptors and serotonin receptors, which are key targets for sympathetic nervous system regulation of blood pressure. The significant enrichment of this pathway suggests that IPM-derived ACE inhibitory peptides can not only act on the peripheral RAAS, but also participate in central blood pressure regulation by modulating the sympathetic nervous system and the release of various vasoactive substances, which expands the understanding of the antihypertensive mechanism of food-derived peptides (Grassi & Ram, 2016 [47]). Although the calcium signaling pathway is not among the top-ranked pathways in terms of enrichment significance, it is a key downstream functional pathway mediating the effects of multiple upstream regulatory signals (including the neuroactive ligand–receptor pathway and RAAS). The contraction and relaxation of vascular smooth muscle cells are directly determined by intracellular calcium concentration, which is the final executive link of vascular tone regulation. Gao et al. (2018) [48] confirmed that natural active substances can promote vasodilation by reducing intracellular calcium concentration in vascular smooth muscle cells. Therefore, the calcium signaling pathway is an important functional link connecting upstream target regulation and final blood pressure phenotype, and its role cannot be ignored. The significant enrichment of this pathway in this study suggests that IPM-derived peptides may exert antihypertensive effects by regulating calcium channel activity and improving vascular smooth muscle tone. The significant enrichment of the calcium signaling pathway in this study suggested that IPM-derived peptides may exert antihypertensive effects by regulating calcium channel activity. The focal adhesion pathway participates in the interaction between cells and the extracellular matrix and is closely related to the vascular remodeling process mediated by MMP2/MMP9 (Souilhol et al., 2020 [49]), further validating the conclusion that improving vascular remodeling is an important antihypertensive mechanism of IPM-derived ACE inhibitory peptides.

#### 3.6.3. Molecular Docking Analysis

Molecular docking enables the simulation of binding modes between ligands and receptors. A lower (more negative) binding free energy indicates stronger ligand–target affinity, with binding energies < −5 kcal/mol generally considered indicative of stable binding (Kumar et al., 2023 [50]). In the present study, molecular docking was employed to validate the ACE inhibitory potential of the eight novel peptides and to elucidate the structural basis underlying their multi-target regulatory effects. All docking simulations were performed using the energy-minimized conformations of the peptides; the reliability of the docking protocol for all eight receptors had been verified via native ligand redocking assays (RMSD < 2.0 A, details provided in Materials and Methods).

##### Molecular Docking of Bioactive Peptides with the Core Target ACE

Molecular docking was conducted with human ACE as the receptor. The results demonstrated that all eight peptides exhibited favorable binding affinity toward ACE, with binding free energies ranging from −6.9 to −10.7 kcal/mol, all satisfying the threshold for stable binding.

**Figure 7:**
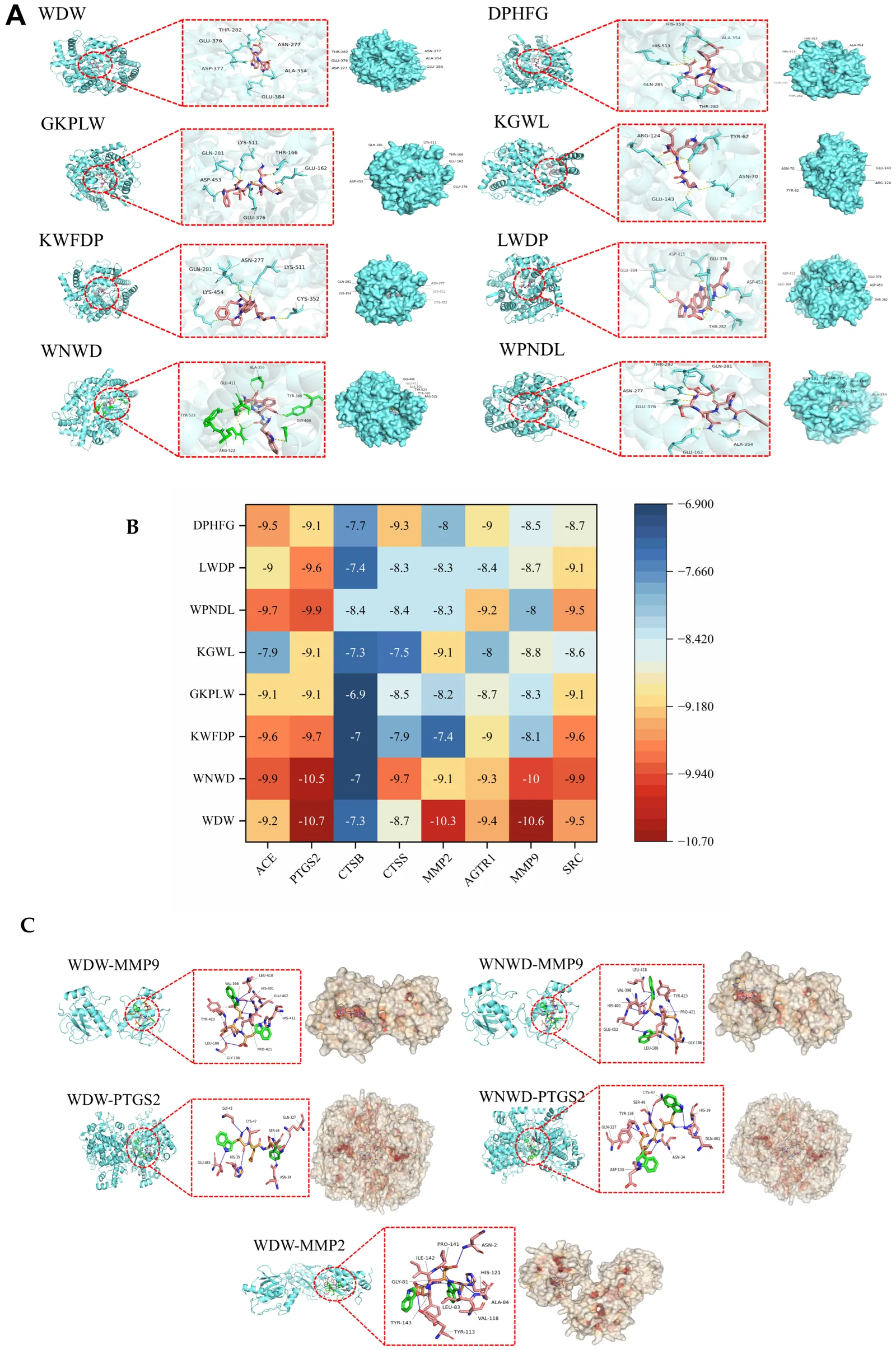
Molecular docking analysis. (**A**) Binding mode visualization of eight novel IPM-derived ACE inhibitory peptides with human angiotensin-converting enzyme. Peptides are presented in stick mode, and the ACE active pocket is shown in surface mode. (**B**) Heatmap of binding free energies from 64 molecular docking simulations between eight IPM-derived ACE inhibitory peptides and eight core antihypertensive targets. The values in the heatmap represent binding free energy in kcal/mol; more negative values indicate stronger binding affinity between the peptide and the target. (**C**) Binding mode visualization of high-affinity peptide–target complexes with binding free energy ≤ −10 kcal/mol, including representative complexes of WDW-MMP9, WNWD-MMP9, WDW-PTGS2, and WDW-MMP2.

Among these, peptide WNWD displayed the strongest binding affinity to ACE (−9.9 kcal/mol). In terms of binding mechanism, WNWD stably occupies the hydrophobic active pocket of ACE through a hydrogen bond network formed with multiple polar residues in the S1 and S2 sub-pockets, as well as a salt bridge interaction with positively charged residues at the pocket entrance. This competitive binding mode can effectively block natural substrates from accessing the catalytic center, thus exerting ACE inhibitory activity (Figure 7A).

##### Systematic Docking Analysis of Bioactive Peptide Candidates with Core Biood Pressure-Related Targets

Systematic molecular docking of the eight peptides with the eight core antihypertensive targets identified via network pharmacology (SRC, MMP9, AGTR1, MMP2, CTSS, ACE, CTSB, and PTGS2) was performed, and the binding free energy results are visualized as a heatmap (Figure 7B). The overall results revealed that the binding free energies of all eight peptides with all eight targets were less than −5 kcal/mol, ranging from −6.9 to −10.7 kcal/mol, thereby providing a structural basis for their multi-target synergistic regulation of blood pressure homeostasis.

Further analysis of core peptides indicated that peptide WDW exhibited the strongest binding affinity toward PTGS2, MMP9, and MMP2, with binding free energies of −10.7, −10.6, and −10.3 kcal/mol, respectively. Peptide WNWD displayed prominent binding affinity toward PTGS2 (−10.5 kcal/mol) and SRC (−9.9 kcal/mol). Notably, PTGS2 is a key mediator of inflammatory responses, and chronic low-grade inflammation is intimately associated with vascular homeostatic imbalance; MMP2 and MMP9 are core effector enzymes mediating vascular remodeling. The capacity of WDW to concurrently and potently bind both PTGS2 and MMP2/9 suggests its potential dual synergistic effects of anti-inflammation and vascular remodeling improvement, indicating that WDW is also a promising candidate peptide for further in-depth research. Additionally, all eight peptides exhibited favorable binding affinities toward targets including AGTR1, CTSB, and CTSS, further corroborating their multi-target synergistic mode of action.

Taking complexes with binding free energy ≤ −10 kcal/mol as representatives, the binding modes of core peptides WDW and WNWD with key targets were visually analyzed. For MMP9, both peptides stably occupy the enzyme’s active cleft: they form hydrogen bonds with key residues in the catalytic center to interfere with catalytic function, and simultaneously form hydrophobic interactions with residues around the pocket to enhance binding stability, thus obstructing substrate binding. For PTGS2, both WDW and WNWD bind to the arachidonic acid substrate channel region through hydrogen bonding and salt bridge interactions, which may inhibit the synthesis of inflammatory mediators by blocking substrate access. For MMP2, WDW stably binds within the active pocket via hydrogen bonding and hydrophobic interactions, thereby inhibiting its hydrolytic activity on extracellular matrix components (Figure 7C).

It should be objectively acknowledged that molecular docking results serve as reference indicators for in vitro activity screening. The trends in ACE inhibitory rates of the peptides may not be entirely concordant with the ranking of binding free energies, due to the influence of peptide solubility, conformational flexibility and other factors in actual reaction systems. The in vivo activity and underlying mechanisms of action require further validation via molecular dynamics simulations and animal experiments in subsequent studies.

### 3.7. In Vitro ACE Inhibitory Activity of the Synthetic WNWD Peptide

Among the eight candidate peptides, WNWD was selected for chemical synthesis and in vitro activity validation for the following reasons: First, WNWD exhibited the strongest binding affinity to the direct research target ACE (binding free energy: −9.9 kcal/mol), which is most closely related to the core objective of ACE inhibitory peptide development. Second, although WDW showed lower binding free energy to individual targets such as PTGS2 and MMP9, its predicted water solubility and gastrointestinal stability were inferior to those of WNWD based on in silico prediction results. Third, the tetrapeptide WNWD has a moderate length, high synthesis yield and low preparation cost, which is conducive to subsequent expanded preparation and application research. Comprehensive consideration led to the selection of WNWD as the core peptide for experimental validation.

**Figure 8:**
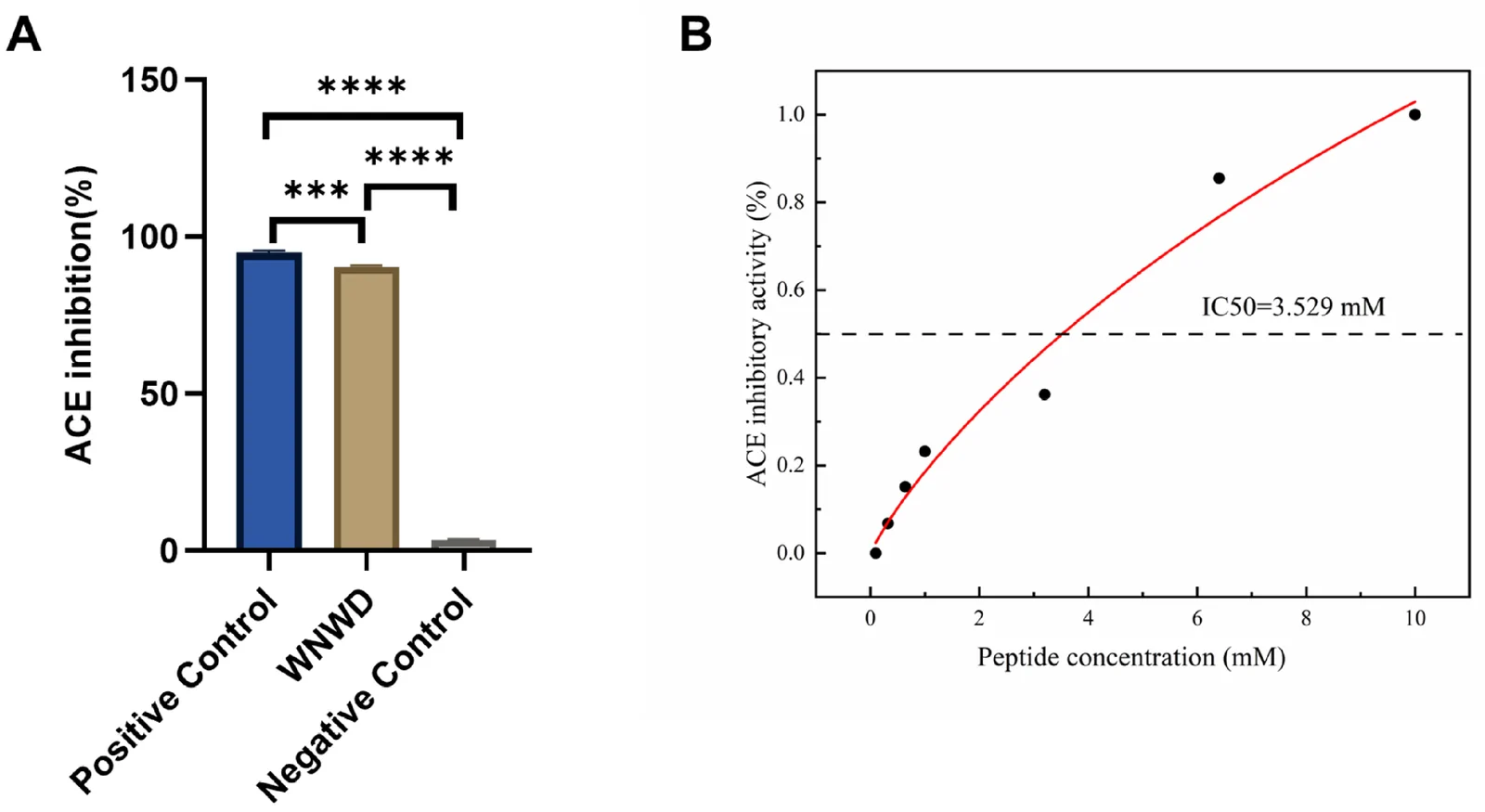
In vitro ACE inhibitory activity validation of synthetic WNWD peptide. All data are expressed as mean ± standard deviation from three independent biological replicates. (**A**) ACE inhibitory rates of captopril (positive control, 10 mM), deionized water (negative control) and synthetic WNWD peptide at 10 mM concentration; (**B**) dose–response curve and half-maximal inhibitory concentration (IC_50_) value of synthetic WNWD peptide against ACE. *** and **** indicates an extremely significant difference between the two indicated groups (*p* < 0.001 and *p* < 0.0001).

The IC_50_ value of WNWD against ACE was determined to be 3.529 mM (Figure 8B). As a positive control, the IC_50_ value of captopril measured under the same experimental conditions was 0.043 μM (Figure 8A), indicating that the inhibitory activity of WNWD is still far lower than that of the synthetic ACE inhibitor drug. Compared with previously reported plant-derived ACE inhibitory peptides, the activity of WNWD is at a moderate level: the IC_50_ values of ACE inhibitory peptides derived from soybean meal, walnut meal and rapeseed meal mostly range from 0.5 mM to 5.0 mM (Sitanggang et al., 2021; Meng et al., 2024; Duan et al., 2023 [3,7,8]), which is close to the results of this study. As a plant-derived tetrapeptide, WNWD has moderate activity, but it has the advantages of wide raw material sources, high biosafety and low preparation cost, and still has good application potential in the field of dietary intervention functional foods.

## 4. Conclusions

This study established a complete technical workflow for the discovery and multi-dimensional characterization of novel ACE inhibitory peptides from *Idesia polycarpa* meal protein, and systematically elucidated their underlying antihypertensive mechanisms. Ultrasound-assisted extraction was confirmed as an effective pretreatment strategy, which can moderately loosen the protein conformation without destroying the core secondary structure, thus improving the subsequent enzymatic hydrolysis efficiency. Through multi-step separation and purification, the low-molecular-weight peptide fraction with the highest ACE inhibitory activity was obtained, and its excellent activity was closely related to the high proportion of hydrophobic and aromatic amino acids. Eight novel food-derived ACE inhibitory peptides were successfully identified, and network pharmacology combined with molecular docking revealed that these peptides exert antihypertensive effects through a multi-target synergistic regulatory mode, involving not only the classic renin-angiotensin system, but also vascular remodeling, inflammatory regulation and neuroendocrine-regulation-related pathways. This multi-target action characteristic distinguishes food-derived bioactive peptides from single-target synthetic antihypertensive drugs, and endows them with potential advantages in long-term dietary intervention for blood pressure homeostasis. The in vitro activity of the core peptide WNWD was verified by solid-phase synthesis, confirming its application potential as a natural ACE inhibitory component. Overall, the findings of this study not only expand the source library of food-derived ACE inhibitory peptides, but also provide a feasible technical path and theoretical support for the high-value and sustainable utilization of *Idesia polycarpa* processing byproducts. Future research can further focus on improving the gastrointestinal stability of the peptides and verifying their in vivo antihypertensive efficacy, so as to promote their application in blood pressure-regulating functional foods.

## Figures and Tables

**Table 1:** Reagent addition volumes for the ACE inhibition assay.

Reagent	Blank Well (μL)	Sample Well (μL)
ACE (0.1 U/mL)	10	10
FAPGG (1 mmol/L)	50	50
Phosphate buffer (80 mmol/L)	50	0
Sample solution	0	50

**Table 2:** LC-MS/MS gradient elution program.

Time (min)	Flow Rate (nL/min)	Mobile Phase B (%)
0.0	300	5
2.0	300	12
20.0	300	30
23.5	300	45
26.5	300	95
30.0	300	95

**Table 3:** Basic information of the eight screened potential antihypertensive peptide sequences.

Number	Sequence	Length	Mass	Core Functional Amino Acids
1	WDW	3	505.20	Trp(×2), Asp
2	WNWD	4	619.24	Trp, Asn, Asp
3	KWFDP	5	691.33	Lys, Trp, Phe, Asp, Pro
4	GKPLW	5	599.34	Gly, Lys, Pro, Leu, Trp
5	KGWL	4	502.29	Lys, Gly, Trp, Leu
6	WPNDL	5	643.30	Trp, Pro, Asn, Asp, Leu
7	LWDP	4	529.25	Trp, Asp, Leu, Pro
8	DPHFG	5	571.24	Phe, Asp, Pro, His, Gly

**Table 4:** Degree value ranking of core antihypertensive targets for CP3-H3 peptides in PPI network.

Number	Gene Symbol	Degree
1	SRC	65
2	MMP9	53
3	AGTR1	47
4	MMP2	40
5	CTSS	36
6	ACE	36
7	PTGS2	34
8	CTSB	34
9	REN	32
10	AGTR2	31

## Data Availability

Data will be made available on request.
